# Bortezomib enhances radiosensitivity in oral cancer through inducing autophagy-mediated TRAF6 oncoprotein degradation

**DOI:** 10.1186/s13046-018-0760-0

**Published:** 2018-04-27

**Authors:** Yuan-Hua Wu, Wun-Syuan Wu, Li-Ching Lin, Chiang-Shin Liu, Sheng-Yow Ho, Bour-Jr Wang, Bu-Miin Huang, Ya-Ling Yeh, Hui-Wen Chiu, Wei-Lei Yang, Ying-Jan Wang

**Affiliations:** 1 Department of Environmental and Occupational Health, College of Medicine, National Cheng Kung University, Tainan, Taiwan; 2 Department of Radiation Oncology, National Cheng Kung University Hospital, College of Medicine, National Cheng Kung University, Tainan, Taiwan; 3 Department of Radiation Oncology, Chi Mei Medical Center, Tainan, Taiwan; 4 School of Medicine, Taipei Medical University, Taipei, Taiwan; 5 Chung Hwa University of Medical Technology, Tainan, Taiwan; 6 Department of Pathology, National Cheng Kung University Hospital, College of Medicine, National Cheng Kung University, Tainan, Taiwan; 7 Department of Radiation Oncology, Chi Mei Medical Center, Liouying, Tainan, Taiwan; 8 Chang Jung Christian University, Tainan, Taiwan; 9 Department of Occupational and Environmental Medicine, National Cheng Kung University Hospital, Tainan, Taiwan; 10 Department of Cosmetic Science and Institute of Cosmetic Science, Chia Nan University of Pharmacy and Science, Tainan, Taiwan; 11 Department of Cell Biology and Anatomy, College of Medicine, National Cheng Kung University, Tainan, Taiwan; 12 Graduate Institute of Clinical Medicine, College of Medicine, Taipei Medical University, Taipei, Taiwan; 13 Division of Nephrology, Department of Internal Medicine, Shuang Ho Hospital, Taipei Medical University, New Taipei, Taiwan; 14 The University of Texas MD Anderson Cancer Center, Houston, TX USA; 15 Department of Biomedical Informatics, Asia University, Taichung, Taiwan; 16 Department of Medical Research, China Medical University Hospital, China Medical University, Taichung, Taiwan

**Keywords:** Oral squamous cell carcinoma, Radiation, TRAF6, Ubiquitination, Autophagy

## Abstract

**Background:**

Oral squamous cell carcinoma (OSCC) is a malignant tumor that may occur anywhere within the oral cavity. The survival rate of OSCC patients has not improved over the past decades due to its heterogeneous etiology, genetic aberrations, and treatment outcomes. We investigated the role of tumor necrosis factor receptor-associated factor 6 (TRAF6) in OSCC cells treated with bortezomib (a proteasome inhibitor) combined with irradiation (IR) treatment.

**Methods:**

The effects of combined treatment in OSCC cells were investigated using assays of cell viability, autophagy, apoptosis, western blotting, and immunofluorescence staining. The ubiquitination of proteins was analyzed by immunoprecipitation. Stable knockdown of TRAF6 in OSCC cells was constructed with lentivirus. The xenograft murine models were used to observe tumor growth.

**Results:**

We found synergistic effects of bortezomib and IR on the viability of human oral cancer cells. The combination of bortezomib and IR treatment induced autophagic cell death. Furthermore, bortezomib inhibited IR-induced TRAF6 ubiquitination and inhibited TRAF6-mediated Akt activation. Bortezomib reduced TRAF6 protein expression through autophagy-mediated lysosomal degradation. TRAF6 played an oncogenic role in tumorigenesis of human oral cancer cells and oral tumor growth was suppressed by bortezomib and IR treatment. In addition, OSCC patients with expression of TRAF6 showed a trend towards poorer cancer-specific survival when compared with patients without TRAF6 expression.

**Conclusions:**

A combination of a proteasome inhibitor, IR treatment and TRAF6 inhibition could be a novel therapeutic strategy in OSCC.

**Electronic supplementary material:**

The online version of this article (10.1186/s13046-018-0760-0) contains supplementary material, which is available to authorized users.

## Background

Oral squamous cell carcinoma (OSCC) is a major subtype of head and neck carcinoma with many pathological differences from cancers found at other sites in the head and neck region and is one of the most widely prevalent cancers throughout the world [1]. OSCC accounts for over 90% of malignant neoplasms of the oral cavity. Its mortality rate has remained largely unchanged over the past decade, with a 5-year survival rate under 50% [2]. However, the molecular and cellular mechanisms underlying the pathogenesis of OSCC are relatively poorly understood. Surgical resection is feasible for OSCC patients, but it is not effective for late-stage metastatic tumors; thus, adding chemotherapy, radiotherapy or both (chemoradiotherapy, CRT) as adjuvant or as definitive treatment are acceptable modalities [3]. Cancer therapy has increasingly focused on novel treatment strategies combining radiotherapy and chemotherapy. Within the context of CRT, the optimal dose for OSCC irradiation has not been clearly defined. To diminish the damage to normal tissue, treatment with chemical modifiers as radiosensitizers in combination with lower dose irradiation (IR) may augment its overall therapeutic efficacy [3, 4]. Recently, the anti-cancer drug bortezomib, the first proteasome inhibitor approved by the U.S. Food and Drug Administration for the treatment of multiple myeloma, has attracted attention for its ability to treat solid tumors alone or in combination with radiotherapy [5, 6]. We have demonstrated previously that a proteasome inhibitor combined with radiation possesses synergistic anti-pancreatic cancer potency both in vitro and in an orthotopic murine model [7]; nevertheless, the effects and the precise mechanism of combined treatment of bortezomib and radiation against OSCC remain unclear.

There are two major protein degradation pathways in eukaryotic cells: the ubiquitin (Ub)-proteasome system (UPS) and the autophagy-lysosome system (hereafter autophagy) [8]. The UPS is a selective proteolytic system in which substrates are recognized and tagged with ubiquitin for degradation. This pathway has essential functions in homeostasis, which include preventing the accumulation of misfolded or deleterious proteins [5, 8]. To ensure appropriate destruction of those proteins that are no longer needed, the components of this system must act in a highly coordinated manner through definitive steps that include polyubiquitylation, deubiquitylation, and degradation of the target protein [9]. In general, ubiquitins are conjugated via a lysine residue at position 48 to target proteins for degradation [10]. Whereas for proteins tagged with lysine 63 (K63)-linked polyubiquitin chains of ubiquitin, instead of being targeted for proteasomal degradation, they alter protein function or localization and thereby regulate signaling activation including receptor endocytosis, protein trafficking, kinase activity and DNA repair [5, 10]. For example, tumor necrosis factor receptor-associated factor 6 (TRAF6), a critical regulator of NF-κB signalling, has been identified as an E3 ligase for K-63-linked polyubiquitination of PKB/Akt. This polyubiquitination promotes membrane recruitment of PKB/Akt and its phosphorylation and activation upon growth factor stimulation [11]. Recently, TRAF6 has also been corroborated to be an oncoprotein involved in cancer development and progression in multiple cancers [12]. Tumor cells overexpressing TRAF6 produce proteins that promote cell growth and survival while meanwhile inhibiting the mechanisms of cell death, and thus, it represents a potential therapeutic target for the treatment of cancer through inhibition of UPS TRAF6-mediated K63-linked polyubiquitination as a means to shift this fine equilibrium towards cell death [5, 13].

Autophagy is a bulk degradative system that uses lysosomal hydrolases to degrade long-lived proteins and damaged or old organelles; it involves membrane formation followed by fusion of the vesicle with lysosomes [14]. Interestingly, autophagy appears to have a dual role in cancer therapy [15]. The proper amount of autophagy promotes cancer cell survival, whereas a high level of autophagy results in autophagic cell death [16]. Whether autophagy has pro-survival or pro-death effects depends on different factors, including cancer cell type/phase, stress context and the microenvironment. Therefore, life or death of the cell is context dependent. Autophagy and UPS were previously thought to be independent of each other in components, action mechanisms, and substrate selectivity. Notably, recent studies suggest that a single proteolytic network in cells comprised of the autophagy and UPS systems functionally cooperate with each other to maintain proteostasis, and the central feature common to both autophagy and UPS is ubiquitination [8]. Ubiquitination can either generate degradation signals on substrates delivered for destruction by proteasomes or lysosomes, or modulate their non-proteolytic processes [17]. The inhibition of the UPS results in the compensatory activation of autophagy, implying there is a crosstalk between autophagy and UPS [8]. It has been reported that bortezomib can induce proteasome independent degradation of TRAF6 in myelodysplastic syndrome [18]. Since TRAF6 is necessary for maintaining the survival of cells, its degradation by bortezomib-induced autophagy contributes to cell death [18]. However, whether TRAF6 plays a critical role in the cross talk between UPS and autophagy in OSCC cancer cells remains undetermined.

In the present study, we investigated the anticancer effect of combined IR and bortezomib treatment on human OSCC cancer cells both in vitro and in a xenograft murine model. The types of cell death, especially autophagic cell death, and the underlying mechanisms, including ubiquitination and phosphorylation of signaling regulators, were examined. In addition, the clinical impact of TRAF6 in oral cancer patients was also investigated.

## Methods

Additional procedures are described in detail in the Additional file 1.

### Irradiation treatment, cell viability and synergistic interaction analysis

Irradiation was performed with 6 MV X-rays using a linear accelerator (Digital M Mevatron Accelerator, Siemens Medical Systems, CA, USA) at a dose rate of 5 Gy/min. An additional 2 cm of a tissue-equivalent bolus was placed on the top of the plastic tissue-culture flasks to ensure electronic equilibrium, and 10 cm of tissue-equivalent material was placed under the flasks to obtain full backscatter. After IR treatment, cells were treated with bortezomib immediately. The treated cells were centrifuged and resuspended with appropriate amount of PBS. For cell viability assay, 20 μl cell suspension was mixed with 20 μl Trypan blue solution (0.4% in PBS). Placing the mixture on a hemocytometer, and the blue-stained cells were counted as nonviable. The effect of the combination treatment was evaluated by the combination index (CI) method using CalcuSyn software (Biosoft), which is based on the median effect model of Chou and Talalay [19]. The experimental data were entered into the CalcuSyn interface and CI values were calculated. CI < 1, CI = 1, and CI > 1 indicate synergism, additive effect, and antagonism, respectively.

### Clonogenic assay

The cells were irradiated using the dosages of 2, 4, 6 or 8 Gy. Bortezomib was added to the cells at concentrations of 25 or 30 nM. The cells were trypsinized and counted. Known numbers of cells were subsequently replated in 6-cm culture dishes and returned to the incubator to allow for colony development. After 2 weeks, colonies (containing≥50 cells) were stained with 0.5% crystal violet solution. The plating efficiency (PE) is the ratio of the number of colonies to the number of cells seeded in the nonirradiated group. Calculation of survival fractions (SFs) was performed using the equation: $\mathrm{SF}=\frac{\kern0.75em \mathrm{colonies}\ \mathrm{counted}\kern0.75em }{\kern0.75em \mathrm{cells}\ \mathrm{seeded}\times \mathrm{PE}\kern0.75em }$.

### Early apoptosis and autophagy detections

Apoptosis was assessed by observing the translocation of phosphotidyl serine to the cell surface, as detected with an Annexin V apoptosis detection kit (Calbiochem, San Diego, CA, USA), according to our previous report [20, 21]. For autophagy analysis, cell staining with acridine orange (Sigma Chemical Co.) was performed according to published procedures [22, 23], adding a final concentration of 1 μg/ml for a period of 20 min. Flow cytometry was used to detect Annexin V-positive cells and acidic vesicular organelles (AVOs).

### Stable knockdown clone selection

For generation of a TRAF6-knockdown stable cell line, SAS cells were transfected with lentiviral vector containing short hairpin RNA (shRNA) purchased from the National RNAi Core Facility located at the Institute of Molecular Biology/Genomic Research Center, Academia Sinica. The clone is identified as TRCN0000007348, which targeted the human TRAF6 transcript sequences, 5′-CGGAATTTCCAGGAAACTATT-3′. We added the lentivirus to cells in a growth media containing 8 μg/*ml polybrene* (MOI = 3). After 16 h post infection, we removed the media and replaced it with media containing puromycin (0.4 μg/ml), and then amplified the cells.

### shRNA transfection

The clone (TRCN0000040123) of shRNA targeting ATG5 was purchased from the National RNAi Core Facility located at the Institute of Molecular Biology/Genomic Research Center, Academia Sinica. We used TransIT-X2 transfection reagent (Mirus Bio Corporation, Madison, WI) to transfect ATG5 shRNA into SAS cells. For 10-cm dish, the total volume of medium and cells per well prior to transfect should be 10 ml. In an eppendorf tube, combined the serum-free medium for 1 ml and plasmid DNA for 10 μl of a 1 μg/μl stock. Added 30 μl TransIT-X2 to the diluted DNA mixture. Pipetted gently to mix completely and incubated at room temperature for 30 min, added total of complex to 10-cm dish for Incubate for 24-48 h. SAS cells were harvested 48 h after shRNA transfection for Western blotting.

### Subcutaneous xenograft in vivo model

Male NOD-SCID mice (5- to 7-weeks-old) were acquired from the National Cheng Kung University Laboratory Animal Center (Taiwan). The animals were housed 5 per cage at 23 ± 2°C with 60% ± 5% relative humidity and subjected to a 12-h light/12-h dark cycle. The animals were adapted to the environment 1 week before the start of the experiments. SAS cells (2 × 10^6^ cells in 0.1 ml of PBS) were subcutaneously inoculated into the right back of the mice. Seven days post injection, the mice were randomized into 5 groups (*n* = 5 for each group): (1) control (99.9% DMSO): mice were injected (intraperitoneally, i.p.) with DMSO. (2) Bortezomib group: i.p. injections with 1 mg/kg Bortezomib twice a week for 3 weeks. (3) IR group: a single dose of 6 Gy IR. (4) Bortezomib + IR: combination therapy with 1 mg/kg Bortezomib twice a week and a single dose of 6 Gy IR at the beginning of the first week. For the TRAF6-knockdown stable clone animal model, the mice were randomized into 4 groups (5 mice per group): normal, control, SAS shTRAF6#1 and SAS shTRAF6#2. Individually, SAS, shTRAF6#1 and shTRAF6#2 cell lines (2 × 10^6^ cells in 0.1 ml of PBS) were subcutaneously inoculated into the right back of the mice and the mice were sacrificed after 3 weeks. Volume estimations were determined using the following formula: $\mathrm{volume}=\frac{\uppi \times \mathrm{width}\times \mathrm{length}\times \mathrm{height}}{6}$. Mouse body weight was measured once per week and was used as an indicator of the systemic toxicity of the treatment. During the experimental period, no deaths occurred in the treatment groups. Mice were sacrificed via CO_2_ exposure. After the mice were sacrificed, the tumor tissues were formalin fixed and paraffin embedded for immunohistochemistry.

### Clinical samples and ethics statement

Clinical tissues were collected from patients who received curative surgery for oral squamous cell carcinoma at Cheng Kung University Hospital, Taiwan. Further adjuvant radio/chemo-radiotherapy was suggested depending on disease status according to head-and-neck cancer treatment guidelines. Histological sections of all cases were reviewed by the pathologist specializing in head and neck cancer, who was blinded to the clinical outcome. The following criteria were used to score the staining: 0: negative (no detectable staining); 1: weakly positive (light yellow staining in cytoplasm); 2: strongly positive (brown cytoplasmic staining). The study was approved by the Institutional Review Board of National Cheng Kung University Hospital (NCKUH-10307004).

### Kaplan-Meier analysis

The cancer specific survival was analyzed with the Kaplan-Meier method by SPSS Ver.17. Univariate analyses of patient and disease characteristics were tested by the log-rank test. Multivariate analysis was calculated by the Cox regression model. *P* values less than 0.05 were considered as statistically significant.

### Statistical analysis

We evaluated the differences in the differences in continuous variables (presented as mean ± standard deviation [SD]) between groups using the two-sample t-test or one-way analysis of variance carrying with a post-hoc Bonferroni test. We performed all statistical analyses using the SPSS 17.0 statistical software (SPSS Inc., Chicago, IL, USA). All statistical tests were performed at a two-sided significance level of 0.05.

## Results

### Synergistic effects of bortezomib and IR on the viability of human oral cancer cells

**Fig. 1:**
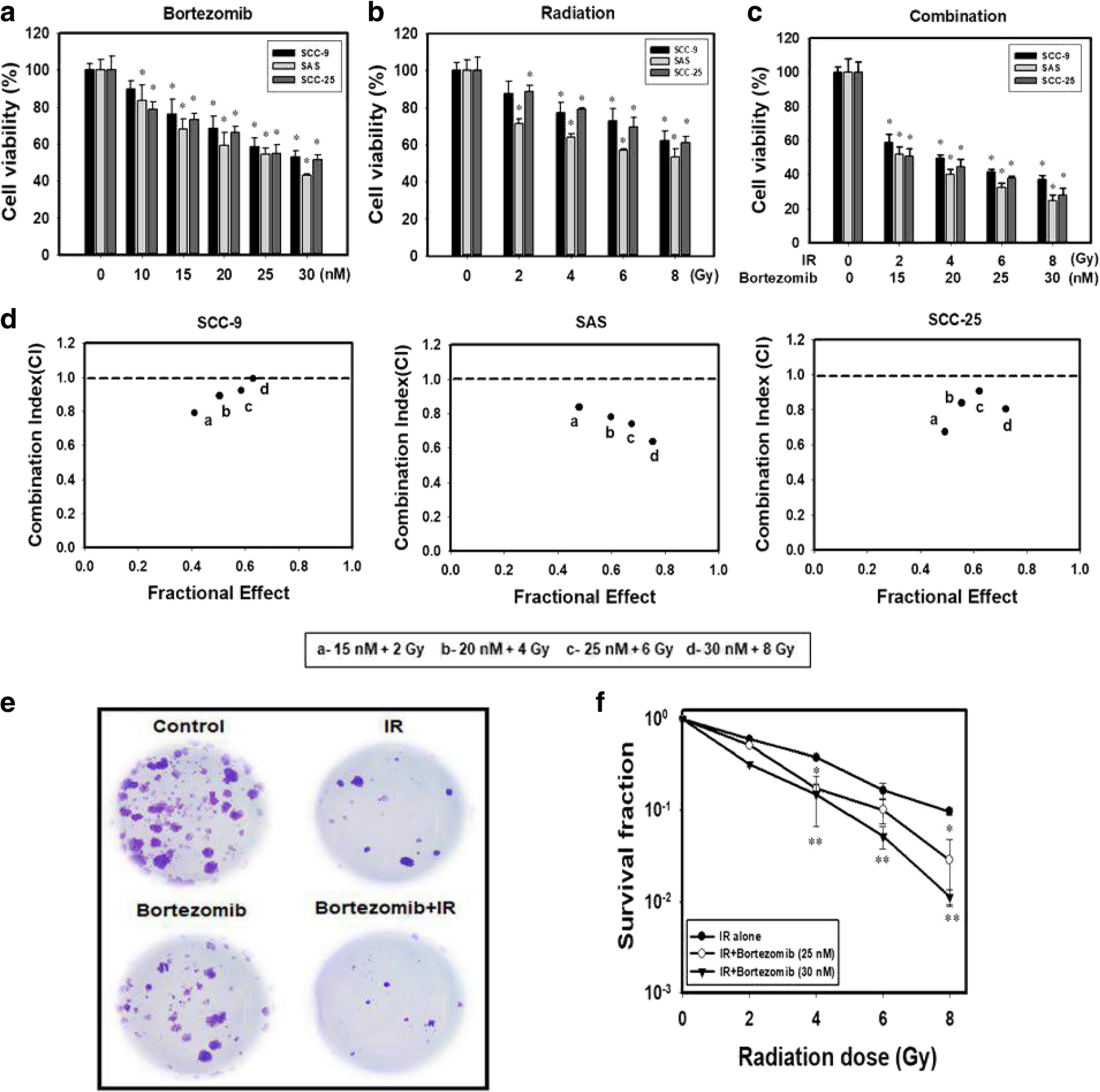
Synergistic effects of bortezomib and IR on the viability of human oral cancer cells. **a** Concentration-dependent effects of bortezomib on the cell viability of SCC-9, SAS and SCC-25 cells. Cells were treated with 0, 10, 15, 20, 25 or 30 nM of bortezomib for 24 h. *p < 0.05, control versus bortezomib. **b** Dose-dependent effects of IR on cell viability of SCC-9, SAS and SCC-25 cells. Cells were treated with 0, 2, 4, 6, or 8 Gy of IR for 24 h. *p < 0.05, control versus IR. **c** Dose-dependent effects of bortezomib combined with IR on cell viability of SCC-9, SAS and SCC-25 cells. *p < 0.05, control versus IR + bortezomib. **d** Combination index (CI) plot of bortezomib, IR, or their combinations on SCC-9, SAS and SCC-25 cells. **e** Bortezomib decreases the clonogenic survival in SAS cells after IR treatment. Cells were stained with crystal violet. **f** Colonies containing> 50 cells were scored as positive. Data are presented as the mean ± standard deviation from three independent experiments. *p < 0.05, IR versus IR + bortezomib (25 nM), **p < 0.05, IR versus IR + bortezomib (30 nM)

First, we investigated the cytotoxic effect of bortezomib and IR either alone or in combination on 3 different human oral cancer cell lines (SCC-9, SAS and SCC25). Both bortezomib and IR inhibited cell viability of human oral cancer cell lines in a concentration- or dose-dependent manner (Fig. 1a and b). In addition, significant enhancement of toxicity was observed in the combined treatment compared with bortezomib and IR treatment alone (Fig. 1c). Furthermore, the combination-index methods developed by Chou and Talalay [19] were used to confirm the observed synergism with IR and bortezomib combined therapy (Fig. 1d). The combined treatment groups displayed synergistic cell killing effects at all tested concentrations (CI < 1) in SCC-9, SAS and SCC25 cells. To further validate whether bortezomib affects radiation sensitivity, radiation dose-response survival curves were determined by clonogenic assay (Fig. 1e and f). The combined treatment with IR and bortezomib resulted in decreased survival fractions compared to cells treated with IR alone. These results indicated that bortezomib treatment clearly radiosensitized the oral cancer cells.

### Combined bortezomib and IR treatment induces autophagic cell death

**Fig. 2:**
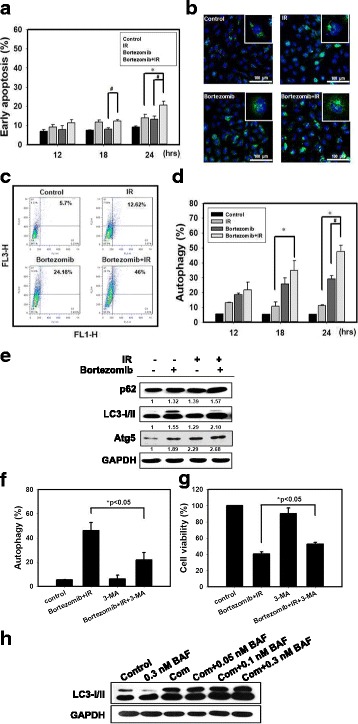
Bortezomib and IR induce autophagy in SAS cells. **a** Measurement of early apoptosis in SAS cells. Early apoptosis detection was measured by flow cytometry with an Annexin V apoptosis detection kit. Cells were treated with 6 Gy of IR or 25 nM of bortezomib for 12, 18, and 24 h. # *p* < 0.05, bortezomib versus IR + bortezomib. * *p* < 0.05, IR versus IR + bortezomib. **b** Confocal immunofluorescence microscopy of LC3 following 24 h treatment with 6 Gy of IR and 25 nM of bortezomib alone or in combination. **c** Measurement of autophagy in SAS cells. Detection of green and red fluorescence in acridine orange-stained cells using flow cytometry. SAS cells were treated with IR (6 Gy) and bortezomib (25 nM) alone or in combination for 24 h. **d** Quantification of AVOs with acridine orange using flow cytometry. Cells were treated with 6 Gy of IR or 25 nM of bortezomib alone or in combination for 12, 18 and 24 h. # *p* < 0.05, bortezomib versus IR + bortezomib. * *p* < 0.05, IR versus IR + bortezomib. **e** Western blot analysis of autophagy-related proteins expression in SAS cells. Cells were treated with 6 Gy of IR and 25 nM of bortezomib alone or in combination for 24 h. **f** Measurement by flow cytometry with AVOs in the absence or presence of 3-methyladenine (3-MA). Cells were pretreated with 3-MA (2 mM) for 1 h before combined treatment (6 Gy of IR and 25 nM of bortezomib) for 24 h. **g** Cytotoxic effects in the absence or presence of 3-MA for 24 h. **h** Western blot analysis of LC3 expression in the absence or presence of bafilomycin A1 (BAF). Cells were pretreated with BAF for 1 h before combined treatment (6 Gy of IR and 25 nM of bortezomib) for 24 h. *p < 0.05, IR + bortezomib + 3-MA versus IR + bortezomib

To assess whether the growth inhibitory effect of combined treatment is related to induction of cell apoptosis, Annexin-V cell surface binding was measured after SAS cell line treatment with bortezomib and/or IR. A low percentage of early apoptosis of cells following treatment with bortezomib or IR has been observed. SAS cells treated with the combined treatment for 24 h showed an increase in the population of early apoptotic cells compared with bortezomib and IR alone (Fig. 2a). Next, we investigated whether combined treatment induced autophagy in SAS cells. Previous studies have demonstrated that bortezomib can induce autophagy in cervical cancer cells [24]. Microtubule-associated protein light chain 3 (LC3) has been used as a specific marker to monitor autophagy [25]. Thus, we applied fluorescence microscopy to determine the percentage of cells with punctate LC3 staining (Fig. 2b). The results showed a significant increase in LC3 immunopositive dots in SAS cells that received combined treatment compared with either bortezomib or IR treatment alone. Furthermore, we analyzed the occurrence of autophagy, which is characterized by the formation of numerous acidic vesicles that are called acidic vesicular organelles (AVOs). To identify the development of AVOs, we used the lysosomo-tropic agent, acridine orange (AO) [26]. AO staining was quantified using flow cytometry (Fig. 2c and d). In SAS cells, we found a significantly increased number of AO-positive cells in the combined treatment group compared to bortezomib or IR alone. We observed increased expression of the autophagy-related proteins LC3-II, Atg5 and p62 following combined treatment (Fig. 2e). 3-Methyladenine (3-MA), an inhibitor of autophagy that is known to inhibit autophagic sequestration [27], suppressed the induction of AVOs in SAS cells after combined treatment (Fig. 2f). In addition, we examined whether 3-MA could alter the cytotoxicity of the combined treatment (Fig. 2g). Compared to the combined treatment group, the pretreatment 3-MA plus combined treatment group displayed significant reduced levels of cytotoxicity. We further confirmed the pro-death role of autophagy in combined treatment-induced cytotoxicity by inhibiting the autophagy flux with bafilomycin A1 (BAF), an inhibitor of autophagosome–lysosome fusion [28]. BAF augmented combined treatment-induced LC3-II accumulation, indicating that autophagy flux was prevented (Fig. 2h). These results showed that combination bortezomib and IR treatment induced autophagic cell death.

### Bortezomib inhibits activation of TRAF6 and NF-κB activity

**Fig. 3:**
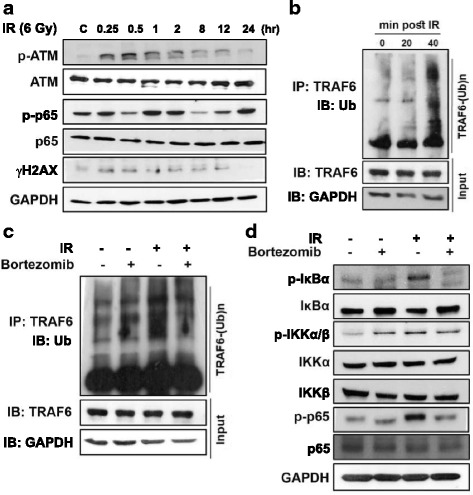
Bortezomib inhibits activation of TRAF6 and NF-κB activity. **a** After the cells were exposed to IR, they were collected and lysed. The expression of phosphorylated ATM, p65 and γH2AX were examined by Western blot analysis. **b** SAS cells were treated with IR, lysed at the times indicated (min), and immunoprecipitated with TRAF6 antibody. IP extracts were analyzed for ubiquitin (Ub) or TRAF6 by immunoblotting. **c** Bortezomib suppressed TRAF6-mediated polyubiquitination by IR. Cells were treated with IR and bortezomib alone or in combination. Cells were treated with 6 Gy of IR or 25 nM of bortezomib for 40 min. **d** Bortezomib inhibited the activation of NF-κB induced by IR. Cells were treated with IR and bortezomib alone or in combination. Cells were treated with 6 Gy of IR or 25 nM of bortezomib for 1 h

In response to genotoxic stress induced by DNA double-stranded breaks (DSBs), the inhibitor of κB kinase (IKK) in the nuclear factor κB (NF-κB) pathway is activated, which can promote cancer progression and increase the resistance of cancer cells to IR or chemotherapeutic drugs [29]. It has been reported that the kinase ataxia telangiectasia mutated (ATM) has an important role in the activation of NF-κB, which stimulates proliferative and antiapoptotic gene programs in response to genotoxic stress [30]. As shown in Fig. 3a, phospho-ATM and phospho-NF-κB contain a 65 kDa subunit (p65) and γH2AX significantly increased in the short term and then decreased in the long term in response to IR. Recent evidence shows that ATM induces activation of NF-κB through the polyubiquitylation of TRAF6 [31]. We found that post-treatment with IR for 40 min induced TRAF6 ubiquitination (Fig. 3b). However, bortezomib inhibited IR-induced TRAF6 ubiquitination (Fig. 3c). We also found that bortezomib suppressed IR-induced phospho-IκBα, p-IKKα/β and p65 (Fig. 3d). These results illustrate that bortezomib can inhibit the activation of TRAF6 and NF-κB activity in SAS cells.

### Bortezomib inhibits TRAF6-mediated Akt activation and reduces TRAF6 protein expression through autophagy-mediated lysosomal degradation

We analyzed the basal expression levels of TRAF6 in human oral keratinocyte (hNOK) cells and 3 human oral cancer cell lines (Additional file 2: Figure S1a). We observed increased TRAF6 levels in the 3 human oral cancer cell lines compared with the hNOK cells. TRAF6 was found to be a direct E3 ligase for Akt and was essential for Akt ubiquitination and phosphorylation upon growth-factor stimulation [11]. We observed decreased TRAF6 protein, Akt and mTOR phosphorylation levels in cells treated with combined bortezomib and IR treatment compared with bortezomib or IR treatment alone (Additional file 2: Figure S1b). Additionally, we found that combined treatment inhibited polyubiquitylation of Akt compared to bortezomib or IR alone (Additional file 2: Figure S1c). To further define the role of TRAF6, we utilized TRAF6 shRNA to inhibit TRAF6 expression in SAS cells. As shown in Additional file 2: Figure S1d, Akt ubiquitination was significantly decreased by TRAF6 shRNA.

**Fig. 4:**
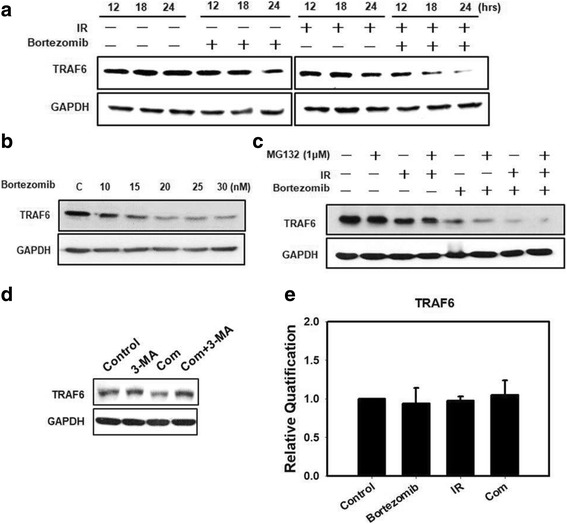
Bortezomib reduces TRAF6 protein expression through autophagy-mediated lysosomal degradation. **a** Western blot analysis of TRAF6 protein expression in SAS cells. Cells were treated with 6 Gy of IR or 25 nM of bortezomib or in combination for 12, 18 and 24 h. **b** Western blot analysis of TRAF6 protein expression in SAS cells. The cells were treated with bortezomib alone with 10, 15, 20, 25, or 30 nM of bortezomib for 24 h. **c** Western blot analysis of TRAF6 protein expression in SAS cells. Cells were treated with 6 Gy of IR or 25 nM of bortezomib or 1 μM of MG132 or in combination for 24 h. **d** Western blot analysis of TRAF6 protein expression in the absence or presence of 3-MA. Cells were pretreated with 3-MA (2 mM) for 1 h before combined treatment (6 Gy of IR and 25 nM of bortezomib) for 24 h. **e** TRAF6 mRNA expression levels were measured by real-time RT-PCR in the indicated cell lines and SAS cells treated with 6 Gy of IR or 25 nM of bortezomib alone or in combination for 24 h

We evaluated TRAF6 expression of bortezomib and IR either alone or in combination treatment. The results showed that bortezomib, IR and combined treatment inhibited TRAF6 expression in a time-dependent manner (Fig. 4a). Furthermore, TRAF6 expression was decreased by bortezomib in a concentration-dependent manner (Fig. 4b). The two major intracellular protein degradation systems are the UPS and autophagy [32]. To determine whether degradation of TRAF6 by bortezomib occurs via UPS, a proteasome inhibitor (MG132) was evaluated. We found that combined treatment with bortezomib and MG132 inhibited TRAF6 expression (Fig. 4c). However, the autophagy inhibitor 3-MA can rescue TRAF6 inhibition (Fig. 4d). In addition, treatment of SAS cells with bortezomib and/or IR treatment resulted in no change in the TRAF6 mRNA levels (Fig. 4e). These results indicated that bortezomib reduced TRAF6 protein expression through autophagy-mediated lysosomal degradation.

### TRAF6 plays an oncogenic role in tumorigenesis of human oral cancer cells and suppression of oral tumor growth by bortezomib and IR treatment

**Fig. 5:**
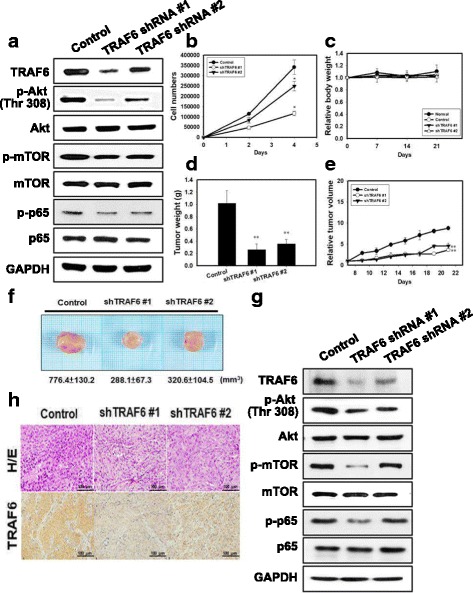
TRAF6 plays an oncogenic role in tumorigenesis of human oral cancer cells in vivo. **a** TRAF6-Akt signaling pathway and p-p65 protein expression in SAS cells transfected with TRAF6 shRNA. **b** Cell growth curve resulting from TRAF6 shRNA in SAS cells. *p < 0.05, versus control. **c** Measurement of body weight in NOD/SCID mice once per week. **d** Measurement of tumor weight of SAS xenograft in NOD/SCID mice. **e** SAS xenograft tumor growth curves in NOD/SCID nude mice. SAS cells silenced with TRAF6 shRNAs were injected into nude mice (*n* = 5 for each group) and monitored for tumorigenesis. ** *p* < 0.01 versus untreated controls. **f** Direct observation of mice with tumors from the control and TRAF6 shRNAs groups. **g** Analysis of the TRAF6-Akt signaling pathway related proteins from tumors transfected with TRAF6 shRNA and grown in NOD/SCID mice by western blot, and (**h**) H/E staining and immunohistochemical staining for analysis of TRAF6-positive cells (brown)

We utilized TRAF6 shRNA to inhibit TRAF6 expression in human oral cancer cells. As shown in Fig. 5a, TRAF6 protein was significantly decreased by TRAF6 shRNAs. Compared with control shRNA, SAS cells transfected with TRAF6 shRNAs had decreased Akt, mTOR and p65 phosphorylation levels compared to control shRNA. We examined whether the inhibition of TRAF6 alters cytotoxic cell proliferation (Fig. 5b). TRAF6 shRNA reduced the viability of SAS cells. We next evaluated the anti-tumor growth effect of TRAF6 shRNAs in vivo. Tumors were induced by the subcutaneous injection of TRAF6-knockdown stable clone cells into SCID immunodeficient mice. We measured the body weight of the mice every week and their tumor volumes every 2 days. The results of this study demonstrated that none of the treatment regimens produced any obvious signs of toxicity in terms of the loss of body weight (Fig. 5c). The tumor volume and tumor weight were reduced in the TRAF6 shRNA groups compared with the control groups (Fig. 5d–f). In tumor tissues, the expression levels of TRAF6, p-Akt, p-mTOR and p-p65 proteins were decreased in the TRAF6 shRNA groups compared with the control groups (Fig. 5g). Histological examination was analyzed by hematoxylin and eosin (H&E) staining (Fig. 5h). The tumors in the TRAF6 shRNA groups were composed of cells with a lower nucleus to cytoplasm ratios than the controls. Furthermore, the TRAF6 level patterns in SAS tumors were examined using IHC staining. TRAF6 expression was decreased in tumors from mice from the TRAF6 shRNA groups compared with the control (Fig. 5h).

Next, SAS cells were injected subcutaneously into SCID mice and allowed to grow for 7 days, prior to randomization of the mice into four groups. During evaluation of the antitumor activity, no apparent changes in mouse body weight were observed in either the treatment or the control group (Additional file 2: Figure S2a). Furthermore, no detectable toxicity was evident by biochemical examination following treatment with bortezomib or IR alone (Additional file 3: Table S1). As shown in Additional file 2: Figure S2b–d, combined treatment of the SAS tumor-bearing mice significantly inhibited tumor growth and tumor weight. Additional file 2: Figure S2e shows an increase of LC3 and decrease of TRAF6 in tumor tissues subjected to combined treatment based on IHC staining data. In addition, the lysates from SCID xenografts were subjected to western blot analysis. Each protein’s expression level in lysates from SCID xenografts was very similar to that in the cultured cell line (Additional file 2: Figure S2f). The combined treatment caused a significant decrease in expression of TRAF6, p-mTOR, p-Akt and p-p65 compared to treatment with bortezomib and IR alone. SAS cells treated with bortezomib and/or IR treatment had increased expression of LC3-II.

### Correlation between TRAF6 expression and prognosis in OSCC patients

**Fig. 6:**
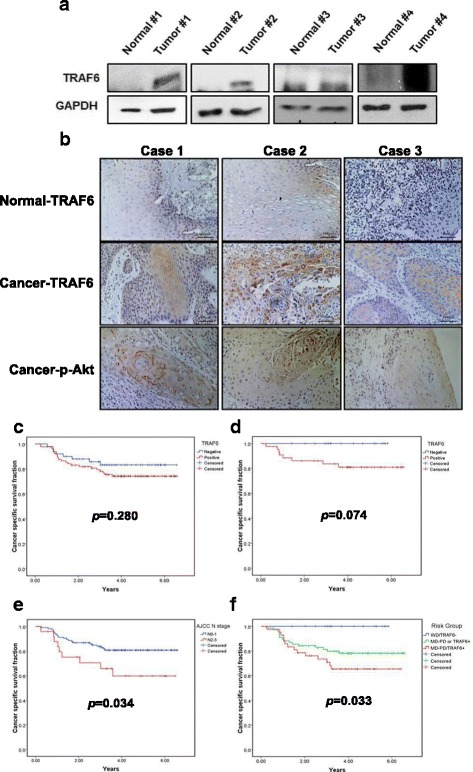
The expression of TRAF6 was elevated in OSCC patients. **a** The protein level of TRAF6 in OSCC samples and paired normal tissues was analyzed by Western blot. **b** The protein level of TRAF6 in human OSCC samples and paired normal tissues was analyzed by IHC. Cancer-specific survival by the Kaplan-Meier method by (**c**) the expression of TRAF6 in all patients (*p* = 0.280), (**d**) the expression of TRAF6 in patients with well differentiated tumors (*N* = 60; *p* = 0.074), (**e**) AJCC N stage (N0–1 vs. N2–3) (*p* = 0.034), and (**f**) Risk groups stratified by differentiation and expression of TRAF6 (*p* = 0.033)

To determine the prognostic significance of TRAF6 expression in OSCC, the tissues were examined by western blot analysis and IHC staining. TRAF6 levels were found to be increased significantly in tumors compared with non-tumor oral tissues (Fig. 6a and b). With a median follow up of 48 months (3–79 months), 139 patients diagnosed with OSCC were included in our analysis. The characteristics of the patients are summarized in Additional file 3: Table S2. Seventeen patients showed strongly positive IHC staining of TRAF6, whereas 71 patients were weakly positive and 51 patients were negative for TRAF6 expression. In univariate analysis, American Joint Committee on Cancer (AJCC) nodal stage 2–3, histology of moderate/poor differentiation, and the addition of radiotherapy to surgery were associated with poorer cancer-specific survival (CSS). The expression of TRAF6 (either strongly positive or weakly positive) in patients showed a trend towards poorer CSS when compared with patients without TRAF6 expression and the effect achieved marginal significance (*p* = 0.074) in patients with well differentiated tumors (Fig. 6c–f). In an analysis using both expression of TRAF6 and differentiation grade as predictor groups of CSS, patients with expression of TRAF6 and moderate/poor differentiation had the worst survival when compared with the other groups (*p* = 0.033, Fig. 6f). In the multivariate analysis, these two risk groups are the only significant predictor of CSS (*p* = 0.043).

## Discussion

The primary objective of combination treatments is to exploit the synergistic effects between the agents, and these optimized combination regimens might help to broaden the applicability of single agent treatment. We provided evidence that combined bortezomib and IR treatment resulted in a synergistic cell-killing effect in SAS human oral cancer cells in vitro and it possessed potent antitumoral activity in a xenograft animal model in vivo (Figs. 1 and Additional file 2: Figure S2). Furthermore, bortezomib alone or in combination with IR caused no detectable toxicity as determined by either biochemical examination or in terms of the loss of body weight (Additional file 2: Figure S2a and Additional file 3: Table S1). A recent review article indicated that the therapeutic effect of radiotherapy can be influenced by regulating autophagy, and the induction of autophagy has become more popular than the use of autophagy inhibitors [33]. Radiation sensitization can be enhanced when the level of autophagy is higher than the tolerance of cells, which can lead to autophagic cell death [34]. It has been shown that an increase in autophagic flux, which is defined as the quantity of degradable material transported from the autophagosome to the lysosome, may be the key factor that modulates autophagy towards cell death [16, 35]. We found that combining bortezomib with IR treatment induced a significant amount of autophagy but only a small amount of apoptosis in SAS, a human oral cancer cell line (Fig. 2). Our in vivo study further demonstrated that the induction of autophagy could be observed in SAS xenograft tumors, in which LC3 was increased following a combined treatment of bortezomib with IR (Additional file 2: Figure S2). Pre-treatment with 3-MA, an inhibitor of autophagy, followed by combined bortezomib with IR treatment of SAS cells, resulted in a significant reduction in cytotoxicity (Fig. 2f). In addition, our current results also showed the combined treatment-induced accumulation of LC3-II in the presence of BAF (Fig. 2h). Thus, the combined treatment of bortezomib with IR increased autophagic flux and induced autophagic cell death in human oral cancer cells. Previous studies have demonstrated that p62 decreased levels can be observed when autophagy is induced [36]. However, data accumulated by us and others have revealed that induction of autophagy was accompanied by increased expression of p62 [20, 37, 38]. After a rapid increase in p62/SQSTM1 expression upon the combination of the phorbol ester PMA and p38MAPK inhibitor SB202190 stimulation, the level of p62 gradually decreased at 24 h [37]. In the present study, we found that the expression levels of the p62 proteins increased with IR and bortezomib alone or in combination at 24 h.

A deeper understanding of the unique functions of autophagy is required for the development of more effective treatments of human cancer [15]. As indicated in our previous study, TRAF6 could serve as a direct E3 ligase for K63-linked ubiquitination of oncogenic Akt, leading to its membrane recruitment and phosphorylation in cells treated with insulin-like growth factor-1 (IGF-1). [11] Since Akt activation is a well-known key event in tumorigenesis, TRAF6 represents a potentially important therapeutic target for human cancers. More recently, we also demonstrated that a proteasome inhibitor (MG132) combined with IR enhanced its anti-pancreatic tumor effects through the induction of autophagy and the downregulation of TRAF6 [7]. Furthermore, a reduced TRAF6 protein level was found in bortezomib-induced autophagy and subsequent cytotoxicity in myelodysplastic syndrome/acute myeloid leukemia [18]. In addition, bortezomib not only affects TRAF6 but also other targets. Previous research has shown that bortezomib inhibits NF-kappaB activity in malignant mesothelioma cells and induces cell cycle blockade and apoptosis [39]. Bortezomib induced mitochondrial apoptosis through regulation of BAK and NOXA [40]. Nonetheless, the potential role of TRAF6 as a therapeutic target in OSCC during chemoradiotherapy combining a proteasome inhibitor with IR has never been reported before. We demonstrated that the TRAF6 expression level in the 3 human oral cancer cell lines was higher than in hNOK, a human normal oral keratinocytes cells (Additional file 2: Figure S1a). Bortezomib alone or in combination with IR inhibited TRAF6-mediated NF-κB/Akt activation through polyubiquitination, and reduced TRAF6 protein levels through autophagy-mediated lysosomal degradation (Figs. 3,4 and Additional file 2: Figure S1). Ubiquitin chains not only generate signals that induce acute degradation of tagged proteins by the proteasome but they also play an essential role for proteins and their associated subcellular organelles by regulating autophagic degradation [41, 42]. We found an increased ubiquitination of TRAF6 and consequent autophagic degradation in OSCC cells treated with bortezomib alone or in combination with IR (Fig. 3). Cells may employ multiple types of heterotypic chain linkages in both autophagy and UPS; however, many questions remain to be answered. For example, what is the molecular decision-making process when the same protein substrates are delivered to the autophagy or UPS? How do cells modulate the activities of the autophagy and UPS in response to various stresses? Understanding the functional relationship between the autophagy and UPS will contribute to the development of therapeutic strategies through modulating proteostasis and removal of pathogenic protein species [8].

In the current study, transfection of TRAF6 shRNA resulted in decreased NF-κB/Akt activation, increased autophagy and reduced viability of SAS cells compared with control shRNA (Figs. 5a and b). Using the TRAF6 knockdown SAS cancer xenograft model system, we were able to demonstrate a decreased tumor growth rate and decreased TRAF6 protein expression in the cancer xenografts when compared with parental SAS cancer xenografts (Fig. 5). The overexpression of TRAF6 oncoprotein has been reported to be associated with increasing tumorigenicity and metastasis of esophageal squamous cell carcinoma in vivo [43, 44]. Tao et al. also found that high TRAF6 expression levels had significantly poorer survival when compared with those with low levels (*P* < 0.05) in 135 patients with colon cancer [45]. However, the clinical impact of TRAF6 in oral cancer patients has seldom being investigated. In our current study, risk groups defined by expression of TRAF6 and level of differentiation were the only predictors of cancer specific survival of OSCC patients. Therefore, TRAF6 is potentially a prognostic marker of OSCC, especially in patients with well differentiated tumors.

## Conclusions

**Fig. 7:**
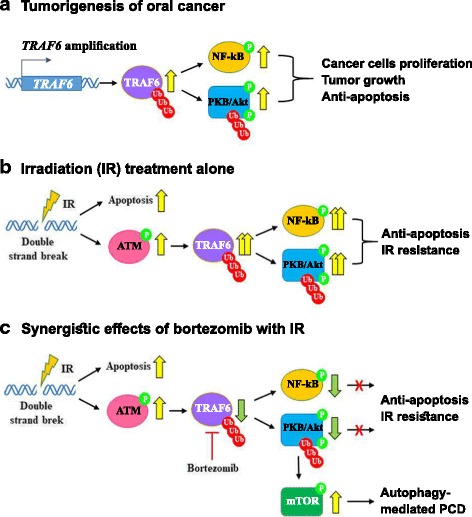
The role of TRAF6 in OSCC cells treated with combined bortezomib and IR treatment. **a** TRAF6-mediated polyubiquitination causes cell proliferation, tumor growth and anti-apoptosis during tumorigenesis of oral cancer. **b** IR treatment alone induces apoptosis. However, IR also induces phosphorylation of ATM and then increases TRAF6 polyubiquitination and phosphorylation of NF-kB, eventually leading to anti-apoptosis and IR resistance. **c** Bortezomib inhibits IR-induced TRAF6 polyubiquitination. Furthermore, bortezomib inhibits TRAF6-mediated Akt activation and induces autophagy-mediated programmed cell death (PCD)

In summary, our results suggest that bortezomib enhanced radiosensitivity through suppression of radiation-induced TRAF6-NF-κB signaling activation. In addition, the combined treatment enhanced the therapeutic efficacy of oral cancer cell lines by downregulating the TRAF6-Akt signaling pathway (Fig. 7). The expression of TRAF6 protein was upregulated in OSCC cell lines and clinical tumor tissue samples. Moreover, downregulation of TRAF6 impaired the tumorigenicity of OSCC cells both in vitro and in vivo. Kaplan-Meier survival analysis showed that patients with TRAF6 expression had a higher risk for death. Thus, our current results provide new insight into the biological properties and clinical relevance of TRAF6 in OSCC. TRAF6 could be a promising target for therapeutic strategies against oral cancer. However, further studies are needed because the mechanism linking autophagy and UPS to TRAF6 remains largely unknown. Our results also indicate that a combination of proteasome inhibitor with IR treatment and TRAF6 inhibition may be a potential therapeutic strategy for the treatment of oral cancer.

## Additional files

Additional file 1: Supplementary material and methods. (DOCX 17 kb)

Additional file 2: **Figure S1.** Bortezomib inhibits TRAF6-mediated Akt activation. **Figure S2.** Combined treatment synergistically inhibits tumorigenesis of human oral cancer cells in vivo. (DOCX 2226 kb)

Additional file 3: **Table S1.** Biochemistry tests including GOT, GPT, albumin, BUN, and creatinine. **Table S2.** Patient characteristics and the analysis results of cancer-specific survival in different variables. (DOCX 16 kb)

## Abbreviations

AO: Acridine orange
AVOs: Acidic vesicular organelles
BAF: Bafilomycin A1
IR: Irradiation
LAMP1: Lysosomal-associated membrane protein 1
MAP1LC3/LC3: Microtubule-associated protein 1 light chain 3
OSCC: Oral squamous cell carcinoma
p62: Sequestosome 1
shRNA: Small hairpin RNA
TRAF6: Tumor necrosis factor receptor-associated factor 6
Ub: Ubiquitin
UPS: Ubiquitin-proteasome system
