# Supplementary material for: Bortezomib enhances radiosensitivity in oral cancer through inducing autophagy-mediated TRAF6 oncoprotein degradation
Source: J Exp Clin Cancer Res. 2018 Apr 27;37:91. doi: 10.1186/s13046-018-0760-0 (PMC5921410; doi:10.1186/s13046-018-0760-0)
Supplement: Supplementary file 1 — Supplementary material and methods. (DOCX 17 kb) [file 13046_2018_760_MOESM1_ESM.docx]

**Supplementary material and methods**

**Cell culture**

The human oral cancer cell lines SCC-9 (ATCC CRL-1629) and SCC-25 (ATCC CRL-1628) were purchased from the American Type Culture Collection (ATCC), and SAS (JCRB0260) was purchased from the Japanese Collection of Research Bioresources (JCRB). The cells were maintained in 1:1 mixture of Dulbecco's Modified Eagle's Medium (DMEM) and Ham's F-12 Nutrient Mixture (Life Technology) supplemented with antibiotics containing 100 U/ml penicillin, 100 mg/ml streptomycin (Gibco BRL, Grand Island, NY) and 10% fetal bovine serum (Caisson Labs, Logan, UT). The primary human oral keratinocyte hNOK was obtained from Dr. D.B. Shieh (Institute of Oral Medicine in Medical College, National Cheng Kung University, Tainan, Taiwan), and was maintained in Keratinocyte Serum-Free Growth Medium (KSFM) (Life Technology) All cells were incubated in a humidified atmosphere containing 5% CO_2_ at 37°C. Exponentially growing cells were detached using 0.05% trypsin-EDTA (Gibco BRL, Grand Island, NY) in medium.

**Immunofluorescence and confocal microscopy**

The cells were cultured on coverslips. After treatment, the cells were fixed in 4% paraformaldehyde and blocked with 1 % BSA for 30 min. This was followed by incubation with a specific antibody against LC3 (Cell Signaling Technology, Ipswich, MA, USA), TRAF6 (GeneTex, Irvine, CA) and LAMP-1 (Novus Biologicals, CO, USA) for 1 h. After washing, the cells were labeled with a DyLight™ 488-conjugated affinipure goat anti-rabbit IgG (Jackson Immuno-Research Laboratories, PA, USA) and rhodamine (TRITC)-conjugated affinipure goat anti-mouse IgG (Jackson Immuno-Research Laboratories, PA, USA) for 1 h. Finally, the cells were stained with DAPI (300 nM) and washed, sealed by mounting a coverslip with an appropriate stabilizing mounting agent, and examined under a confocal microscope (Carl Zeiess LSM780, Instrument Development Center, NCKU).

**RNA extraction and real-time RT-qPCR analysis**

The RNA was extracted using a TRIzol reagent (Invitrogen, USA), and then reverse-transcribed using random primers at 70 °C for 10 min, chilled on ice to stop the reaction, and then we added 5×buffer, 10 mM dNTP and 0.1 DTT at 42 °C for 5 min, followed by adding Super scrip II at 42 °C for 60 min and then 72 °C for 15 min, and chilled on ice, according to the manufacturer instructions. Total RNA (2 μg) was reverse transcribed and the resulting cDNA was amplified using the specific TaqMan gene expression assays for TRAF6 and GAPDH (Applied Biosystems, USA) (assay ID, Hs00371512_g1, Hs02758991_g1, respectively). The quantitative PCRs were performed on the StepOne Plus Real Time PCR System (Applied Biosystems, USA) in accordance with the manufacturer's instructions. Assays were performed in triplicate with appropriate nontemplate controls. Scale-up data were normalized to GPADH expression levels. Quantification of relative expression (reported as arbitrary units [a.u.]) was performed using the 2-ΔΔCt relative quantification method. The quantitative PCR data presented a variability coefficient of Ct that was always lower than 2% of the mean values.

**Western blotting and immunoprecipitation-western blotting**

SAS cells were lysed, separated by 6-15% SDS-PAGE gel, transferred to nitrocellulose membrane and then blocked with skim milk. GAPDH expression indicated the protein loading control. The following antibodies were used for IP or immunoblotting (IB): anti-GAPDH(ab8245) was obtained from Abcam (Cambridge, MA, USA); anti-LC3(AP1802a) was obtained from Abgent (San Diego, CA, USA); anti-TRAF6(1660-1), mTOR(GTX101557), phospho-mTOR(GTX79009), AKT 1/2/3(GTX121937), histone H2AX (GTX127340) and ATM (GTX70103) were obtained from GeneTex (Irvine, CA); anti-p62/SQSTM1(PM045) was obtained from MBL (Nagoya, Japan); anti- phospho(Thr308)-Akt (9275), IκBα(4814), phospho-IKKα/β(2697), IKKα(2682), IKKβ(2678), phospho-NF-κB p65(3033) were obtained from Cell Signaling Technology (Ipswich, MA, USA); anti-UBB (10201-2-AP) was obtained from Proteintech Group (Chicago, IL), anti-phospho(S32/S36)-IkBα(AF4809) was obtained from R&D Systems (Minneapolis, MN, USA); anti-LAMP-1(NBP2-25155) was obtained from Novus Biologicals (CO, USA). Total SAS cells were lysed and pre-cleared with protein G plus/protein agarose (Merck Millipore, Darmstadt, Germany) affect at 4 °C for 2 h. After 2 h later, centrifugation and take the supernatant liquid, added primary antibodies in immunoprecipitation buffer affect at 4 °C overnight. Next, protein G plus/protein agarose were added for 1 h, beads were washed three times with immunoprecipitation buffer. After the mix of 5× sample dye, heated the samples at 95 °C for 10 min, place it in the ice for 5-10 min, and put through to Western blotting analysis.

**Histological analysis**

Mouse and human formalin-fixed tissue samples were coated on glass slides, deparaffinized in xylene, and rehydrated throught 100%, 95%, 75% ethanol. Next microwaved in sodium citrate buffer (pH 6.0) to recovered the antigen, slides were incubated with 3% H_2_O_2_/Methanol at room temperature to block endogenous peroxidase activity. The slides were incubated with an anti-TRAF6 (GeneTex, Irvine, CA), anti-phospho (Ser473)-Akt (GeneTex, Irvine, CA), anti-LC3 (Abgent, San Diego, CA, USA), and STARR TREK Universal HRP detection kit (Biocare medical, Concord, CA). Finally, the slides were counterstained with hematoxylin.
