# Supplementary material for: Bortezomib enhances radiosensitivity in oral cancer through inducing autophagy-mediated TRAF6 oncoprotein degradation
Source: J Exp Clin Cancer Res. 2018 Apr 27;37:91. doi: 10.1186/s13046-018-0760-0 (PMC5921410; doi:10.1186/s13046-018-0760-0)
Supplement: Supplementary file 2 — Figure S1. Bortezomib inhibits TRAF6-mediated Akt activation. Figure S2. Combined treatment synergistically inhibits tumorigenesis of human oral cancer cells in vivo. (DOCX 2226 kb) [file 13046_2018_760_MOESM2_ESM.docx]

**Supplementary Figures**

**
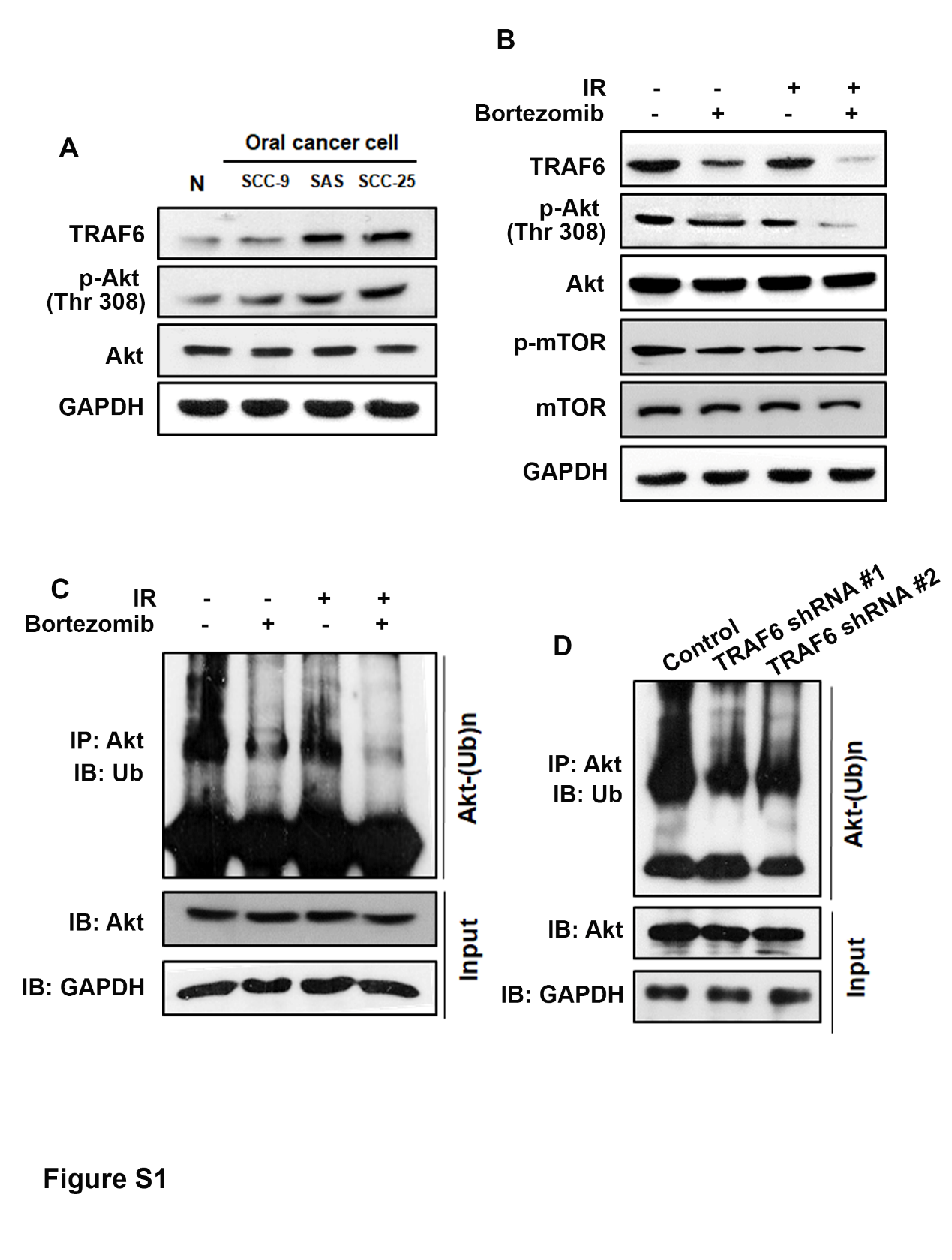
**

**Figure S1.**

Bortezomib inhibits TRAF6-mediated Akt activation. (a) Western blot analysis of Akt-TRAF6 signaling pathway protein expression in hNOK, SCC-9, SAS and SCC-25 cells. N: normal oral cells (human oral keratinocytes cells, hNOK) (b) Western blot analysis of TRAF6-Akt signaling pathway protein expression in SAS cells. Cells were treated with IR and bortezomib alone or in combination. Cells were treated with 6 Gy of IR or 25 nM of bortezomib for 24 h. (c) Cells were treated with 6 Gy of IR or 25 nM of bortezomib or in combination for 24 h before whole cell extract collection. Equal amounts of proteins in each cell extract were subjected to immunoprecipitation (IP) using anti-Akt. The immunoprecipitates were analyzed by Western blotting using anti-polyubiquitin. (d) SAS cells were transfected with TRAF6 shRNA. Equal amounts of proteins in each cell extract were subjected to immunoprecipitation (IP) using anti-Akt and analyzed by Western blotting using anti-polyubiquitin.


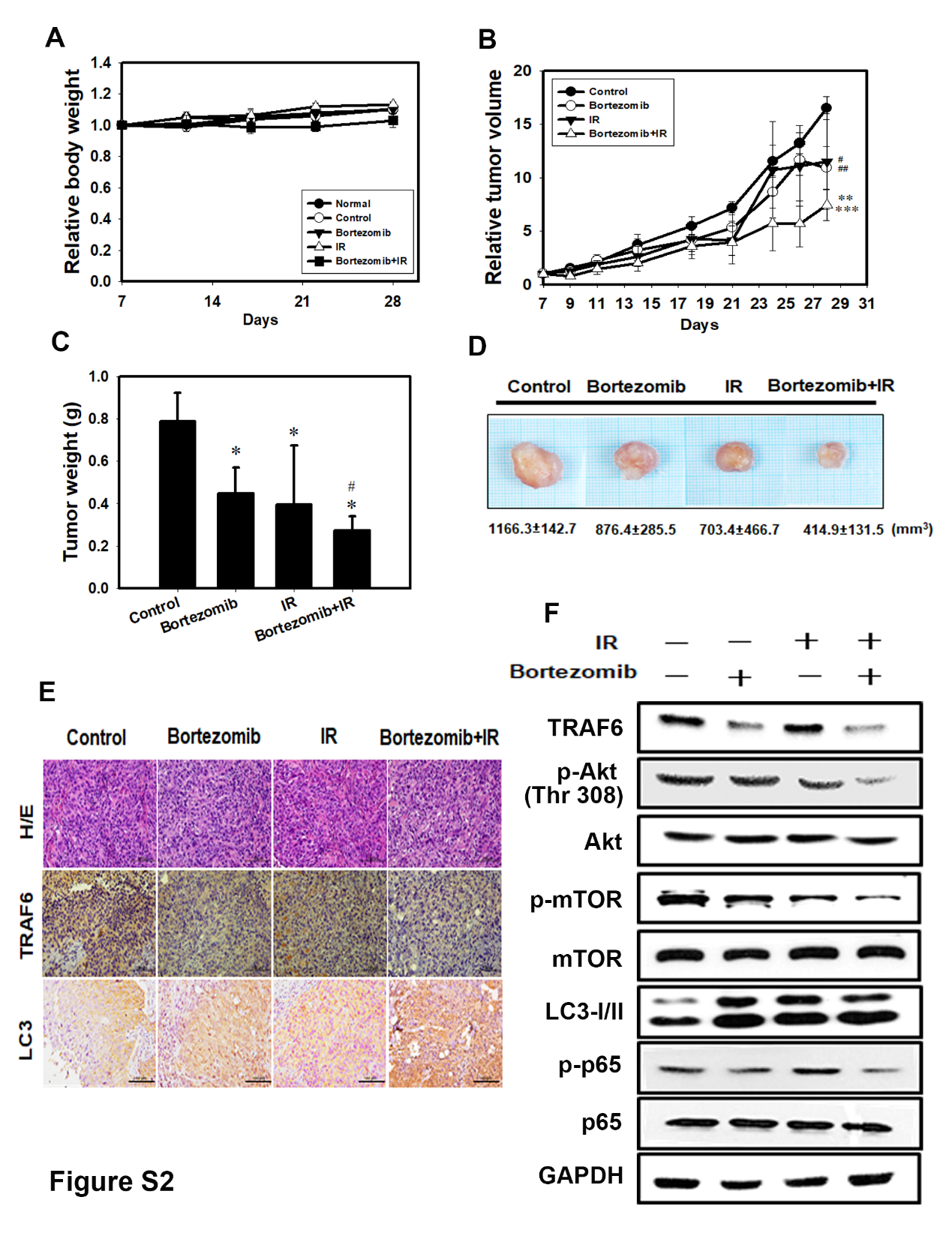


**Figure S2.**

Combined treatment synergistically inhibits tumorigenesis of human oral cancer cells *in vivo.* (a) Measurement of body weight in NOD/SCID mice once per week. (b) SAS xenograft tumor growth curves in NOD/SCID mice. Data are presented as the relative tumor volume normalized to the initial tumor volume measured on Day 0 as a function of time after start of treatment. ^#^ *p* < 0.05, IR versus untreated controls. ^##^ *p* < 0.05, bortezomib versus untreated controls. ** *p* < 0.05, combined treatment versus untreated controls. *** *p* < 0.05 combined treatment versus bortezomib. (c) Measurement of tumor weight of SAS xenografts in NOD/SCID mice. * *p* < 0.05, versus untreated controls. ^#^ *p* < 0.05, bortezomib versus combined treatment. (d) Direct observation of mice with tumors from the control and bortezomib, IR alone or in combination groups. (e) H&E staining as well as immunohistochemical staining for analysis of TRAF6-positive and LC3-positive cells (brown). (f) Western blot analysis of TRAF6, p-Akt, p-mTOR, LC3 and p-p65.
