# Supplementary material for: Bortezomib enhances radiosensitivity in oral cancer through inducing autophagy-mediated TRAF6 oncoprotein degradation
Source: J Exp Clin Cancer Res. 2018 Apr 27;37:91. doi: 10.1186/s13046-018-0760-0 (PMC5921410; doi:10.1186/s13046-018-0760-0)
Supplement: Supplementary file 3 — Table S1. Biochemistry tests including GOT, GPT, albumin, BUN, and creatinine. Table S2. Patient characteristics and the analysis results of cancer-specific survival in different variables. (DOCX 16 kb) [file 13046_2018_760_MOESM3_ESM.docx]

**Supplementary Table 1.** Biochemistry tests including GOT, GPT, albumin, BUN, and creatinine.

|  |  |  | SAS cells | |  |
| --- | --- | --- | --- | --- | --- |
| Item/Group | Normal | Control | Bortezomib | IR | Bortezomib+IR |
| GOT (U/l) | 95.6±23.81 | 97.5±27.98 | 111.2±56.29 | 114.4±44.32 | 97.8±36.91 |
| GPT (U/l) | 17.4±2.19 | 15.83±5.04 | 17.33±2.88 | 15.2±1.92 | 19.6±4.04 |
| ALB (g/dl) | 2.72±0.22 | 2.73±0.14 | 2.69±0.16 | 2.66±0.21 | 2.5±0.32 |
| BUN (mg/dl) | 27.64±0.75 | 27.02±2.1 | 28.85±0.64 | 29.77±3.52 | 26.45±0.96 |
| CRE (mg/dl) | 0.32±0.04 | 0.33±0.05 | 0.37±0.06 | 0.33±0.06 | 0.37±0.05 |

**Supplementary Table 2.** Patient characteristics and the analysis results of cancer-specific survival in different variables.

| **Variables** | **Number** | **Univariate Analysis*** | **Multivariate analysis**** |
| --- | --- | --- | --- |
| **Age** | 24-84 (Median 51) |  |  |
| **Gender** |  | 0.301 | N/A |
| **Male** | 135 |  |  |
| **Female** | 4 |  |  |
| **Smoking** |  | 0.804 | N/A |
| **Yea** | 128 |  |  |
| **No** | 11 |  |  |
| **Alcohol** |  | 0.253 | N/A |
| **Yes** | 105 |  |  |
| **No** | 34 |  |  |
| **AJCC T stage** |  | 0.148 | 0.781 |
| **T1-3** | 97 |  |  |
| **T4** | 42 |  |  |
| **AJCC N stage** |  | 0.034 | 0.456 |
| **N0-1** | 114 |  |  |
| **N2-3** | 25 |  |  |
| **Differentiation** |  | 0.032 | 0.104 |
| **Well** | 60 |  |  |
| **Moderately**  **/poorly** | 79 |  |  |
| **Treatment type** |  | 0.012 | 0.305 |
| **Surgery only** | 89 |  |  |
| **Surgery+RT** | 50 |  |  |
| **TRAF6 status** |  | 0.280 | 0.151 |
| **Negative** | 51 |  |  |
| **Positive** | 88 |  |  |
| **Risk group** |  | 0.033 | 0.043 |
| **I (WD+ TRAF6-)** | 17 |  |  |
| **II (MD/PD orTRAF6+)** | 77 |  |  |
| **III(MD/PD + TRAF6+)** | 42 |  |  |

*Calculated by Kaplan-Meier method. ** Calculated by Cox-Proportional Model. Abbreviations: RT: radiotherapy; WD: well differentiation; MD: moderately differentiated; PD: poorly differentiated
